# Reviewers in 2023

**DOI:** 10.1098/rsbl.2024.0070

**Published:** 2024-03-27

**Authors:** David Beerling

**Affiliations:** *Editor-in-Chief*

Since 2017 *Biology Letters* has recognized the contributions made by reviewers who evaluated our manuscripts in our annual article of thanks. On behalf of the Editorial Board and editorial staff, I would like to once-again express my sincere gratitude to all our reviewers during 2023. This also includes any co-reviewers, who may have had their first experience of peer-reviewing though our manuscripts.

We are aware that most reviewers value recognition of their efforts by the journal and through services, such as Publons, which is integrated with Biology Letters. To provide an opportunity for recognition, we also list the names of all reviewers (unless they have requested otherwise). This article is made permanent and citable by its digital object identifier (DOI). We strongly encourage you to quote your contribution when applying for tenure and grants, or other forms of research assessment. Where you have provided it, we have included your ORCID iD, to ensure your contribution can be unambiguously assigned to you.

Your valuable insights that go into each manuscript help to ensure the robustness of the scientific record, and the *Biology Letters* team looks forward to working with you all again in the future.

Abegglen L https://orcid.org/0000-0003-3607-3001
Abernathy V
  Adkins-Regan E
  Aguilar L
  Ahern O https://orcid.org/0000-0003-4787-7582
Akcay C https://orcid.org/0000-0003-0635-9586
Albery G https://orcid.org/0000-0001-6260-2662
Alhajeri B https://orcid.org/0000-0002-4071-0301
Alza Leon L
  Anders S
  Anderson SAS
  Andrews J
  Anton S
  Arostegui M https://orcid.org/0000-0002-9313-9487
Asher R https://orcid.org/0000-0002-4434-9074
Ashour D
  Atchoi E
  Ausprey I https://orcid.org/0000-0002-7127-2746
Authier M
  Avarguès-Weber A https://orcid.org/0000-0003-3160-6710
Avosani S https://orcid.org/0000-0001-8605-5237
Aznar FJ
  Bahlai C https://orcid.org/0000-0002-8937-8709
Bahreini R
  Baird E https://orcid.org/0000-0003-3625-3897
Bakker J https://orcid.org/0000-0001-6670-1887
Balunas M
  Baniel A https://orcid.org/0000-0001-7142-5864
Barbadilla A
  Barber I https://orcid.org/0000-0003-3955-6674
Barclay K
  Barnett J https://orcid.org/0000-0001-9789-4132
Barton N
  Bath E https://orcid.org/0000-0002-2988-8061
Bäumler F https://orcid.org/0000-0001-5484-1671
Bautista N https://orcid.org/0000-0003-0634-0842
Beard KC https://orcid.org/0000-0002-6279-9837
Beck S https://orcid.org/0000-0001-6426-1603
Becker D https://orcid.org/0000-0003-4315-8628
Becker J https://orcid.org/0000-0003-4168-8350
Becker T
  Begall S https://orcid.org/0000-0001-9907-6387
Benton M https://orcid.org/0000-0002-4323-1824
Bentz E
  Bernal M
  Berthaume M https://orcid.org/0000-0003-1298-242X
Bertolucci C https://orcid.org/0000-0003-0252-3107
Bianco G https://orcid.org/0000-0002-6144-2959
Bianucci G https://orcid.org/0000-0001-7105-0863
Bignell J
  Bisconti M
  Bitter M https://orcid.org/0000-0001-7607-2375
Blackburn D
  Blackstone N
  Blake K https://orcid.org/0000-0003-4834-4120
Blumstein D https://orcid.org/0000-0001-5793-9244
Bonier F https://orcid.org/0000-0001-7245-0244
Booth D
  Botero C https://orcid.org/0000-0003-0955-2795
Botting J
  Bourke A https://orcid.org/0000-0001-5891-8816
Boyd R
  Brace S https://orcid.org/0000-0003-2126-6732
Brelsfoard C
  Bressac C
  Brestic M
  Bromage T https://orcid.org/0000-0002-9843-7993
Brown C
  Brumm H https://orcid.org/0000-0002-6915-9357
Brunner J
  Bruns E https://orcid.org/0000-0003-4184-0922
Buchler N
  Buchmann S
  Budd G https://orcid.org/0000-0001-9007-4369
Burness G
  Burraco P https://orcid.org/0000-0002-9007-2643
Burton R
  Burzyński A
  Büsse S https://orcid.org/0000-0002-1657-7950
Butler A
  Butts I
  Buzatto B https://orcid.org/0000-0002-2711-0336
Cacho N
  Cahill AE
  Caldwell M https://orcid.org/0000-0002-2377-3925
Calhim S
  Camp A
  Campbell D https://orcid.org/0000-0001-8996-5463
Camplisson E
  Canoine V https://orcid.org/0000-0003-2272-0634
Carcaud J https://orcid.org/0000-0003-3672-9963
Carli G
  Carlson J
  Carlson N https://orcid.org/0000-0002-7785-1247
Carlson R
  Carotenuto F
  Carver S https://orcid.org/0000-0002-3579-7588
Casacci L
  Casagrande S https://orcid.org/0000-0002-4264-8062
Castelan F
  Castro-Rondon D
  Cerda X
  Certel S https://orcid.org/0000-0003-2039-2657
Chapple D
  Charruau P
  Chén O https://orcid.org/0000-0002-5696-3127
Chen P
  Cheney K https://orcid.org/0000-0001-5622-9494
Chiel E https://orcid.org/0000-0002-7146-3219
Chow PKY https://orcid.org/0000-0002-8208-592X
Chowdhury S
  Chowdhury M
  Christofides S https://orcid.org/0000-0002-3806-3416
Civitello D https://orcid.org/0000-0001-8394-6288
Claidiere N https://orcid.org/0000-0002-4472-6597
Clifton I https://orcid.org/0000-0003-3615-2447
Collins A
  Colombelli-Negrel D https://orcid.org/0000-0002-9572-1120
Convey P https://orcid.org/0000-0001-8497-9903
Coombes M
  Coombs E
  Cooper T https://orcid.org/0000-0001-5038-9366
Corn K
  Corsaro D
  Courtiol A https://orcid.org/0000-0003-0637-2959
Crego R
  Croijmans I
  Crowe-Riddell J https://orcid.org/0000-0003-2794-2914
Csata E https://orcid.org/0000-0003-2564-9706
Cubaynes S https://orcid.org/0000-0002-3935-9825
Czaczkes T https://orcid.org/0000-0002-1350-4975
da Silva J https://orcid.org/0000-0001-5631-5421
Dam H https://orcid.org/0000-0001-6121-5038
Daniele P
  Darden S https://orcid.org/0000-0002-2567-7902
Dardente H http://orcid.org/0000-0001-7209-5940
Darveau C-A
  de Flamingh A
  de Medeiros B https://orcid.org/0000-0003-1663-668X
De Meester G https://orcid.org/0000-0001-7051-9957
de Vries D
  Debruyne R
  Dechaume-Moncharmont F-X
  Deeming DC https://orcid.org/0000-0002-9587-6149
Deksne G
  Del-Claro K
  Demolin D
  Denef V
  Díaz López B
  Dietemann V
  Dikakou S-E
  Dini P
  DiNuzzo M
  Dion-Côté A-M
  Dodson P
  Doligez B
  Dorsey O
  Doublet V
  Douhard M https://orcid.org/0000-0001-9422-7270
Doums C
  Drake J https://orcid.org/0000-0003-4646-1235
Du W-G https://orcid.org/0000-0002-1868-5664
Dudley R https://orcid.org/0000-0003-3707-5682
Dugas MB
  Dunbar D
  Duncan A https://orcid.org/0000-0002-6499-2913
Dunn R
  Duro S
  Edelaar P https://orcid.org/0000-0003-4649-6366
Eens M
  Egeland J
  Elbroch LM https://orcid.org/0000-0002-0429-4179
Eleftherianos I https://orcid.org/0000-0002-4822-3110
Ellis JT
  Ellis R http://orcid.org/0000-0002-3117-0075
Elvidge C
  Englund G
  Erickson P https://orcid.org/0000-0001-8420-995X
Espinosa J https://orcid.org/0000-0003-0780-2762
Evans L
  Everaert G
  Faith T
  Fargevieille A https://orcid.org/0000-0003-0934-3563
Farine D https://orcid.org/0000-0003-2208-7613
Farrell T
  Feldheim K
  Fenton B https://orcid.org/0000-0001-9761-5412
Ferreira E https://orcid.org/0000-0003-0497-6118
Ferrón H https://orcid.org/0000-0003-2254-8424
Field J https://orcid.org/0000-0003-0663-4031
Fields C https://orcid.org/0000-0002-4812-0744
Filatov D
  Filion A https://orcid.org/0000-0003-1198-3017
Finkbeiner S https://orcid.org/0000-0001-8525-6141
Firestein S
  Fish F
  Fisher S https://orcid.org/0000-0002-3132-1996
Fitzgerald E
  Fleischmann P
  Fleishman L https://orcid.org/0000-0002-2309-7496
Flynn J
  Fobert E https://orcid.org/0000-0001-5020-0615
Forstmeier W https://orcid.org/0000-0002-5984-8925
Foster J https://orcid.org/0000-0002-4444-2375
Fowler-Finn K https://orcid.org/0000-0003-2951-3283
Franco D https://orcid.org/0000-0002-0731-9511
Fraser K
  Fraune S
  Freitak D https://orcid.org/0000-0001-8574-0531
Fricke C https://orcid.org/0000-0002-0691-6779
Friedman M https://orcid.org/0000-0002-0114-7384
Friesen C https://orcid.org/0000-0001-5338-7454
Gagne F
  Galen S
  Galmes J
  Gamelon M https://orcid.org/0000-0002-9433-2369
Gangloff E https://orcid.org/0000-0002-9074-8009
Garbelotto M
  Garcia LF
  Gardner A https://orcid.org/0000-0002-1304-3734
Gargaglioni Batalhão LH
  Garitano Zavala Á
  Gasiorowski L
  Gavriilidi I https://orcid.org/0000-0003-1035-1931
Gérard M https://orcid.org/0000-0002-2485-0662
Gerken A
  Gerry S
  Gill S https://orcid.org/0000-0002-4628-8922
Gilmour K https://orcid.org/0000-0001-9056-4704
Giroud S https://orcid.org/0000-0001-6621-7462
Goel V
  Golubović A
  Gonzalez Betancourt VH https://orcid.org/0000-0002-4146-1634
Gorsich E https://orcid.org/0000-0002-3017-0540
Goumas M https://orcid.org/0000-0001-9338-2412
Graham P
  Gray J https://orcid.org/0000-0001-9079-7962
Greco G https://orcid.org/0000-0003-3356-7081
Greenhill S https://orcid.org/0000-0001-7832-6156
Gregory S
  Greiman S
  Griffith S https://orcid.org/0000-0001-7612-4999
Gross T https://orcid.org/0000-0002-1356-6690
Grundy E
  Grunst A https://orcid.org/0000-0001-5705-9845
Guarneri A https://orcid.org/0000-0002-9162-2731
Gutiérrez J https://orcid.org/0000-0001-8459-3162
Haag E
  Hampton J
  Hao Y
  Harper E https://orcid.org/0000-0002-1092-3867
Hartfelder K
  Hebets E https://orcid.org/0000-0002-9382-2040
Hedenström A https://orcid.org/0000-0002-1757-0945
Helantera H https://orcid.org/0000-0002-6468-5956
Hendry T
  Henneberg M
  Henry L https://orcid.org/0000-0002-3130-4643
Henshaw J https://orcid.org/0000-0001-7306-170X
Hensley N https://orcid.org/0000-0003-0681-0808
Hetem R
  Hill G
  Ho S
  Hobern D
  Hobson K
  Hodgson D
  Hofmann H https://orcid.org/0000-0002-3335-330X
Holleley C https://orcid.org/0000-0002-5257-0019
Holowka N https://orcid.org/0000-0003-0593-7524
Hörberg T
  Hosokawa T
  Howard SR https://orcid.org/0000-0002-1895-5409
Hsu B-Y https://orcid.org/0000-0002-3799-0509
Huber N https://orcid.org/0000-0002-2371-5645
Huey R https://orcid.org/0000-0002-4962-8670
Hughes N
  Hulse S https://orcid.org/0000-0003-1268-0202
Hurley L
  Hurtado-Monzón A
  III AE
  Ioannou C https://orcid.org/0000-0002-9739-889X
Iriarte-Diaz J https://orcid.org/0000-0003-3566-247X
Isberg S
  Isojunno S https://orcid.org/0000-0002-2212-2135
Jamie G https://orcid.org/0000-0002-2766-7687
Jani M
  Jastroch M
  Jensen P https://orcid.org/0000-0001-5491-0649
Jernigan C https://orcid.org/0000-0002-1877-953X
Jo I
  Johansson F https://orcid.org/0000-0002-2302-2603
Johnson J
  Jolles J https://orcid.org/0000-0001-9905-2633
Käfer H
  Kalcounis-Rueppell M
  Kaltenpoth M https://orcid.org/0000-0001-9450-0345
Kaňuch P
  Kapralov M
  Katsougia E
  Kear B https://orcid.org/0000-0002-3128-3141
Kekäläinen J https://orcid.org/0000-0001-6303-6797
Kelu J
  Kenrick P https://orcid.org/0000-0002-3626-5460
Kirchengast S
  Kishida T
  Klamser P
  Klein N
  Klemme I https://orcid.org/0000-0001-9757-7356
Klug C https://orcid.org/0000-0002-4099-7453
Kobak J
  Konarzewski M https://orcid.org/0000-0001-7428-6521
Korsten P
  Kosicki J
  Kotrschal A https://orcid.org/0000-0003-3473-1402
Krams I https://orcid.org/0000-0001-7150-4108
Krasheninnikova A https://orcid.org/0000-0001-8566-8277
Krol E
  Kronauer D https://orcid.org/0000-0002-4103-7729
Krueger-Hadfield S
  Kučera M
  Kuntner M
  Kuo C-Y https://orcid.org/0000-0002-8533-9358
Kurtz J https://orcid.org/0000-0002-7258-459X
Kurvers R https://orcid.org/0000-0002-3460-0392
Kusumi K
  Lachance A
  Lachaud J-P
  LaDue C https://orcid.org/0000-0002-3224-0397
Lampert K https://orcid.org/0000-0002-0383-6132
Langerhans R https://orcid.org/0000-0001-6864-2163
Langwig K
  Lanz Mendoza H
  Laplagne DA
  Laskowski K https://orcid.org/0000-0003-1523-9340
Lavaud J
Lawson S https://orcid.org/0000-0002-7628-8042
Lazzari C https://orcid.org/0000-0003-3703-0302
Leahy B
  Leinonen T
  Leitao E https://orcid.org/0000-0002-6041-9511
Leleu A https://orcid.org/0000-0002-2943-8183
Lemaire J
  Leongómez JD https://orcid.org/0000-0002-0092-6298
Lessig E
  Li J https://orcid.org/0000-0001-7947-0950
Li Q
  Lifjeld JT https://orcid.org/0000-0002-9172-9985
Lloyd V https://orcid.org/0009-0006-8467-0133
LIu Z https://orcid.org/0000-0003-1084-9449
Llaurens V
  Long B
  Longrich N https://orcid.org/0000-0002-9586-8907
Lopez A
  Lopez D
  Lopez-Marcano S
  López-Torres S
  Lovas-Kiss A
  Lovern M
  Lynch V
  MacDougall-Shackleton S https://orcid.org/0000-0001-8518-765X
Machado F
  Machado-Rodrigues A
  Madsen T https://orcid.org/0000-0002-0998-8372
Manas F
  Mankin R
  Manns M https://orcid.org/0000-0001-8860-9504
Marcantonio M https://orcid.org/0000-0003-3896-2355
Marcus J https://orcid.org/0000-0001-6605-3437
Mari L https://orcid.org/0000-0002-7373-0587
Mariette M https://orcid.org/0000-0003-0567-4111
Markham M
  Marlatt V
  Marshall H
  Martin-Ordas G https://orcid.org/0000-0002-5221-9181
Masek P
  Masson F
  Matsuzawa T https://orcid.org/0000-0002-8147-2725
Matthiopoulos J https://orcid.org/0000-0003-3639-8172
Maturana C https://orcid.org/0000-0002-4427-8093
Maxwell E https://orcid.org/0000-0002-6032-6251
Mazzamuto MV
  McAlister S
  McCabe B
  McConnel G
  McCullough J https://orcid.org/0000-0002-7664-3868
Meccariello R
  Mejia A
  Mendoza H
  Meredith R
  Merilä J https://orcid.org/0000-0001-9614-0072
Mesoudi A https://orcid.org/0000-0002-7740-1625
Michaud M https://orcid.org/0000-0001-7075-9862
Micheletta J https://orcid.org/0000-0002-4480-6781
Mila B
  Milne G
  Miroliubov A
  Mirth C https://orcid.org/0000-0002-9765-4021
Miszkiewicz J https://orcid.org/0000-0002-9769-2706
Mitchell D
  Mitkus M
  Miyatake T https://orcid.org/0000-0002-5476-0676
Modregger P
  Momeni B https://orcid.org/0000-0003-1271-5196
Mondanaro A
  Mondet F
  Montgomery T
  Morash A
  Moreno-Rueda G https://orcid.org/0000-0002-6726-7215
Morita M https://orcid.org/0000-0002-0170-1226
Mortimer B
  Mossman H
  Mousel M
  Moyano M
  Muheim R
  Munday P
  Murphy J
  Nadeau N https://orcid.org/0000-0002-9319-921X
Nager R
  Nascimento F
  Naug D https://orcid.org/0000-0001-9252-8037
Naciri M
  Navarro E
  Navarro N
  Nazari F
  Neate-Clegg M https://orcid.org/0000-0001-9753-6765
Nedoluzhko A https://orcid.org/0000-0001-7040-0892
Nemec P https://orcid.org/0000-0003-0277-0239
Nicieza A https://orcid.org/0000-0003-4062-569X
Nieri R
  Nietlisbach P https://orcid.org/0000-0002-6224-2246
Nitta J
  Niven J https://orcid.org/0000-0001-7786-5254
Noble D https://orcid.org/0000-0001-9460-8743
Nunney L https://orcid.org/0000-0002-4315-3694
Ocklenburg S
  O'Donnell S https://orcid.org/0000-0002-6338-7148
Oefele M https://orcid.org/0009-0008-4152-6320
Ogihara N https://orcid.org/0000-0002-1697-9263
O'Hanlon N
  O'Hara R https://orcid.org/0000-0001-9737-3724
Ornelas-García CP
  Osorio DC
  Osvath M https://orcid.org/0000-0002-7873-0930
Oufiero C
  Ożarowska A
  Ożgo M
  Padget R https://orcid.org/0000-0002-6607-6388
Palaoro A https://orcid.org/0000-0002-8629-0728
Panasiuk A
  Pandey A
  Pap P https://orcid.org/0000-0002-3659-7684
Papenbrock J
  Pardo Sanchez J
  Parker J
  Parthasarathy B
  Pavón-Vázquez C
  Pearson D
  Pečnerová P
  Pedretti G
  Peeters M
  Pekar S https://orcid.org/0000-0002-0197-5040
Penalba J https://orcid.org/0000-0001-6549-6885
Penn H https://orcid.org/0000-0002-3692-5991
Pepke M https://orcid.org/0000-0002-6280-1829
Pepperberg I https://orcid.org/0000-0001-9486-7323
Perelmuter J https://orcid.org/0000-0001-9785-8211
Pérez L
Pérez-García A
  Peris D https://orcid.org/0000-0003-4074-7400
Perotti MA
  Perrichot V
  Peter C
  Pfennig D https://orcid.org/0000-0002-1114-534X
Pigeon G
  Pike T https://orcid.org/0000-0002-6942-0498
Pinseel E
  Piou C
  Pires M
  Piscopo M
  Platt B
  Podos J https://orcid.org/0000-0002-2546-7999
Polet D
  Popescu S
  Porter C https://orcid.org/0009-0003-7883-286X
Porter W
  Pozo Romero MI
  Prakash A https://orcid.org/0000-0001-6922-2806
Pritchard D
  Pulgar J
  Pyenson N https://orcid.org/0000-0003-4678-5782
Qian R
  Quertermous H https://orcid.org/0000-0002-0069-9304
Raia P https://orcid.org/0000-0002-4593-8006
Ramm S https://orcid.org/0000-0001-7786-7364
Rasmussen A
  Raven J https://orcid.org/0000-0002-2789-3297
Rawlence N https://orcid.org/0000-0001-6968-6643
Reeve H
  Reid S
  Reinke A
  Reinke B
  Rhodes J https://orcid.org/0000-0002-4871-4331
Richards M https://orcid.org/0000-0003-0071-2350
Riddell E
  Riede T https://orcid.org/0000-0001-6875-0017
Riedel A https://orcid.org/0000-0002-8291-4425
Rindermann H https://orcid.org/0000-0002-5137-8922
Roberts A
  Robillard T https://orcid.org/0000-0002-2177-9549
Robins H
  Robinson E https://orcid.org/0000-0003-4914-9327
Rodriguez A https://orcid.org/0000-0001-7882-135X
Rodríguez-Seijo A https://orcid.org/0000-0003-4868-3069
Ronget V
  Rönnegård L
  Rosati A https://orcid.org/0000-0002-6906-7807
Rosengrave P https://orcid.org/0000-0001-8421-3094
Rosenheim J
  Rosenthal G
  Rossi G
  Rosvall K https://orcid.org/0000-0003-3766-9624
Roth T
  Rowe M
  Rozaimi M https://orcid.org/0000-0001-6631-8677
Ruffier F https://orcid.org/0000-0002-7854-1275
Rugani R https://orcid.org/0000-0001-5294-6306
Runnegar B
  Rypstra A
  Sagi A
  Sakata J https://orcid.org/0000-0003-2285-9310
Sandoval L
  Sandoz JC https://orcid.org/0000-0002-5423-9645
Sauer M
  Schal C https://orcid.org/0000-0001-7195-6358
Scharsack J
  Scheiner R https://orcid.org/0000-0002-7515-4253
Scheyer T https://orcid.org/0000-0002-6301-8983
Schilthuizen M
  Schmidt-Lebuhn A http://orcid.org/0000-0002-7402-8941
Schmitt D
  Schmitt T
  Schmoll T https://orcid.org/0000-0003-3234-7335
Schneider J https://orcid.org/0000-0001-8523-7354
Schneider-Crease I
  Schowanek S
  Schuh A
  Schulz A https://orcid.org/0000-0001-8007-5157
Schweitzer M
  Scott-Samuel N
  Searcy W https://orcid.org/0000-0002-1969-0561
Sekar M
  Sepp T https://orcid.org/0000-0002-8677-7069
Servedio M
  Seymour R https://orcid.org/0000-0002-3395-0059
Shahmohamadloo R https://orcid.org/0000-0002-5373-9808
Sharbrough J
  Shawkey M https://orcid.org/0000-0002-5131-8209
Sheehan M https://orcid.org/0000-0002-3949-7873
Shepherd L http://orcid.org/0000-0002-7136-0017
Sheridan J
  Short S https://orcid.org/0000-0002-1074-8982
Shrader A https://orcid.org/0000-0002-6451-6132
Siddiqui R https://orcid.org/0000-0001-9646-6208
Simon M
  Singer M
  Smallegange I
  Smith AC
  Smith D
  Smith H
  Smith LD
  Sobral G
  Socha J https://orcid.org/0000-0002-4465-1097
Soliz J
  Song H
  Sørensen JG
  Spellman G
  Spence M
  Spiecker R https://orcid.org/0000-0003-3368-0885
Spool J https://orcid.org/0000-0002-9130-9526
Srygley R
  Stavenga DG
  Steiger S https://orcid.org/0000-0002-9714-5665
Stewart J https://orcid.org/0000-0001-6851-2289
Stier A https://orcid.org/0000-0002-5445-5524
Streicher T https://orcid.org/0000-0003-1315-7042
Studds C
  Stynoski J https://orcid.org/0000-0002-9132-8567
Šulc M https://orcid.org/0000-0002-7866-1795
Sun B https://orcid.org/0000-0002-7318-6059
Sun X-Y https://orcid.org/0000-0003-3770-1997
Sustaita D https://orcid.org/0000-0001-9932-909X
Suter H https://orcid.org/0000-0002-5415-6030
Sutherland J https://orcid.org/0009-0007-2638-5681
Szabo JK https://orcid.org/0000-0002-8786-1887
Szekely T https://orcid.org/0000-0003-2093-0056
Tabery J
  Taborsky M https://orcid.org/0000-0002-1357-4316
Takano O https://orcid.org/0000-0002-3819-4299
Teixeira C
  Ten González S https://orcid.org/0000-0002-7284-6916
Theobald J https://orcid.org/0000-0001-7648-3772
Thompson González N
  Thompson K https://orcid.org/0000-0002-1487-3254
Thomson J
  Tichit P
  Tierney S https://orcid.org/0000-0002-8812-6753
Titon S https://orcid.org/0000-0002-1351-6546
Todorova B https://orcid.org/0000-0002-6684-8800
Tomás G
  Touchon J https://orcid.org/0000-0002-1643-348X
Tragust S https://orcid.org/0000-0002-3675-9583
Treberg J
  Trewick S https://orcid.org/0000-0002-4680-8457
Truchy A
  Trueblood L
  Tryjanowski P https://orcid.org/0000-0002-8358-0797
Turley S https://orcid.org/0009-0004-2308-1488
Ueno O
  Unal G
  Upchurch P
  Üveges B https://orcid.org/0000-0001-9234-9258
Uwe M
  Vallortigara G https://orcid.org/0000-0001-8192-9062
Valsecchi P
  Van Damme R https://orcid.org/0000-0002-4585-6940
van de Kamp T https://orcid.org/0000-0001-7390-1318
van der Burg C https://orcid.org/0000-0002-2337-5935
van der Kooi CJ https://orcid.org/0000-0003-0613-7633
van Leeuwen C https://orcid.org/0000-0003-2833-7775
van Velzen E
  Vandeleest J https://orcid.org/0000-0001-7880-0700
Vannier J https://orcid.org/0000-0003-0998-1231
Vedder O
  Vega-Trejo R
  Vélez-García JF
  Verhulst S https://orcid.org/0000-0002-1143-6868
Vernesi C
  Vijendravarma R
  Vila-Farré M
  Vinther J https://orcid.org/0000-0002-3584-9616
Viranta S https://orcid.org/0000-0003-4105-530X
Vivekanandhan P
  Vlachos E https://orcid.org/0000-0002-1980-7109
Vogt K https://orcid.org/0000-0002-8763-342X
von Philipsborn A
  Wagner D
  Wagner P
  Wahl R
  Wambiji N
  Wang B
  Wang S https://orcid.org/0000-0002-0496-1728
Warburton E https://orcid.org/0000-0001-8125-2741
Warren D
  Warrier V https://orcid.org/0000-0001-5166-6409
Weaver L
  Weihrauch D https://orcid.org/0000-0002-3218-9093
Weisbecker V https://orcid.org/0000-0003-2370-4046
Weisberg A
  Westerman E https://orcid.org/0000-0002-3575-8298
Wheeler C
  White E https://orcid.org/0000-0002-0768-9555
Whitehead H https://orcid.org/0000-0001-5469-3429
Whiting M https://orcid.org/0000-0002-4662-0227
Wiberg RAW https://orcid.org/0000-0002-8074-8670
Wild G https://orcid.org/0000-0001-7821-7304
Williams H https://orcid.org/0000-0003-0947-6243
Williams T https://orcid.org/0000-0002-6416-9441
Wilson A https://orcid.org/0000-0001-9914-3455
Wilson D
  Wilson J https://orcid.org/0000-0002-9124-4048
Wisnoski N https://orcid.org/0000-0002-2929-5231
Witt C https://orcid.org/0000-0003-2781-1543
Wodowski G
  Wojcik L https://orcid.org/0000-0001-7178-6782
Wojczulanis-Jakubas K
  Wolf F https://orcid.org/0000-0002-0955-8487
Wolff J https://orcid.org/0000-0003-2326-0326
Wolmarans DW
  Wylie D
  Wyneken J
  Xi N
  Young H https://orcid.org/0000-0003-0449-8582
Yu Q https://orcid.org/0000-0002-8665-0383
Zakon H https://orcid.org/0000-0002-3700-4668
Zardus JD
  Zeppelin T
  Zimmer A
  Zuk M

